# The Use of Pepsin in the Local Treatment of Diphtheria and Membranous Croup

**Published:** 1889-01

**Authors:** 


### CULLINGS FROM CONTEMPORARIES.

### THE USE OF PEPSIN IN THE LOCAL TREATMENT OF DIPHTHERIA AND MEMBRANOUS CROUP.

The field for the use of pepsin seems constantly extending with the improvements made in the quality of this agent, and it may now be employed with greater certainty as to results than ever before. The application of pepsin to digest away the membrane in diphtheria and membranous croup is not new, and is more or less commended and resorted to by physicians in the treatment of these diseases.

Naturally, however, its utility depends entirely upon its digestive activity, and on account of the many preparations of pepsin of feeble or no digestive power heretofore at the disposal of physicians the results obtained have been in some cases discouraging.

As to the value of pepsin, however, in these affections when of proper purity and strength, there can be no question. We believe that the recent improvements in pepsin, securing greater purity, strength and permanence (we allude to the pepsin purum in lamellis of Parke, Davis & Co.), will lead to its extensive use in diphtheria and membranous croup, maladies now attended with such grave results even when combatted by the most expert medical care.

is to be hoped, and it is certainly highly probable, that the further study of digestive ferments will lead to the production of a pepsin still more active.

If the false membrane could be easily digested, and there seems no reason why it might not be with a pepsin of high digestive power, we could expect to have fewer cases of interference with respiration and blood poisoning from absorption of septic material, now alas so frequent.

Dr. H. D. Chapin, of New York, has made some interesting experiments in the solution of croupous membrane. He says an alkaline solution, not strong enough to act as an escharotic, had no influence on the membrane, or at most produced but slight softening. Experimenting with trypsin he found that croupous membrane was dissolved in from fifteen to twenty minutes by the spray and by solution, the spray acting a little more rapidly than the solution. A solution of trypsin required five hours to disolve the mucus expectoration from phthisis.

Pepsin solutions have been less used and less experimented with than trypsin, and yet this seems likely to give far better results when it does come into more general use.

A solution of pepsin will disolve croupous membrane outside the body in from fifteen to thirty minutes, acting as well as trypsin, with the very important advantage that it does not require an alkaline solution.

The reaction of the fluids of the mouth and throat in diphtheria is markedly acid, and the great majority of local medicinal applications in general nse are acid, hence the combination of pepsin with acid fluids can be more easily accomplished than the efforts to keep up an alkaline condition for the use of other solvents.

Speaking of solvents in diphtheria, DaCosta says: "The remedy that has done best is a saturated solution of pepsin in the form of a spray."

But a spray is a terror to most children, especially infants, and many practitioners are deterred from using any remedy, however useful, which has to be applied in that form. In my own practice I always use a swab or sponge, on a bent rod, applying the solution of pepsin freely to the diseased surfaces, every one, two or three hours, according to the severity of the case, and quantity of false membrane present.

The objection to the probang may be made that a child does not submit to its use any more gracefully than to that of the spray; but it takes but a moment to make the application, even if force be necessary to accomplish that object, and the effects are more lasting than the spray, necessitating its use much less frequently.

In regard to the form of pepsin, allow me to say that I have used several, and find that those that come in a scale or crystal form, so called, are the most active; the powdered form being difficult of solution and not seeming to possess the energy of the former.

Two years ago I began the use of solutions of pepsin locally, with the same general treatment, since when my cases have made a more rapid, and better recovery than when the same treatment without the pepsin was administered.—Exchange.

